# Home telehealth and paediatric palliative care: clinician perceptions of what is stopping us?

**DOI:** 10.1186/1472-684X-13-29

**Published:** 2014-06-16

**Authors:** Natalie K Bradford, Jeanine Young, Nigel R Armfield, Anthony Herbert, Anthony C Smith

**Affiliations:** 1 Centre for Online Health, University of Queensland, Level 3 Foundation Building Royal, Children’s Hospital, Herston Rd, Herston, Queensland 4029, Australia; 2 Queensland Children’s Medical Research Institute, University of Queensland, Royal Children’s Hospital, Herston Rd, Herston, Queensland 4029, Australia; 3 School of Nursing and Midwifery, University of the Sunshine Coast, Sippy Downs, Queensland 4556, Australia; 4 Paediatric Palliative Care Service, Royal Children’s Hospital, Brisbane, Queensland 4029, Australia

**Keywords:** Palliative care, Paediatric, Home care, Telehealth, Telehospice, Health service

## Abstract

**Background:**

Advances in technology have made the use of telehealth in the home setting a feasible option for palliative care clinicians to provide clinical care and support. However, despite being widely available and accessible, telehealth has still not been widely adopted either in Australia or internationally. The study aim was to investigate the barriers, enablers and perceived usefulness for an established home telehealth program in paediatric palliative care from the perspective of clinicians.

**Methods:**

Semi-structured interviews (n = 10) were undertaken with palliative care clinicians in a tertiary paediatric hospital to identify attitudes to, satisfaction with, and perceived benefits and limitations of, home telehealth in palliative care. Iterative analysis was used to thematically analyse data and identify themes and core concepts from interviews.

**Results:**

Four themes are reported: managing relationships; expectations of clinicians; co-ordination, and the telehealth compromise. Core concepts that emerged from the data were the perceived ability to control clinical encounters in a virtual environment and the need to trust technology. These concepts help explain the telehealth compromise and low utilisation of the home telehealth program.

**Conclusions:**

Effective communication between caregivers and clinicians is recognised as a core value of palliative care. Home telehealth has the potential to provide a solution to inequity of access to care, facilitate peer support and maintain continuity of care with families. However, significant limitations and challenges may impede its use. The virtual space creates additional challenges for communication, which clinicians and families may not intuitively understand. For home telehealth to be integrated into routine care, greater understanding of the nature of communication in the virtual space is required.

## Background

There is increasing social and political pressure for health services to utilise telehealth to provide cost efficient and equitable care across communities [1-3]. The use of video-consultation in the home to provide palliative care, sometimes referred to as telehospice, is one area of significant interest and numerous studies undertaken have investigated the effects for clinicians and patients [4-7]. Video consultation in the home has been used to provide: symptom management; support and advice; continuity of care, and improve equity of access to specialist care [8]. However, uptake resulting in established services has continued to be slow; most projects do not eventuate into an established service [4,9-12]. The reasons for the poor uptake of home video-consultation in palliative care has largely been attributed to organisational factors such as staff ‘readiness’, lack of a clinical champion or financial incentives [11], as well as concerns with the appropriateness of technology being used instead of in person ‘face to face’ visits [13].

At the Royal Children’s Hospital (RCH) in Brisbane Australia, interest in the use of video-consultation in patients’ homes to support paediatric palliative care has been significant and sustained over the last decade. This can be attributed to several factors: 1) the vast geographical size of Queensland, and the dispersion of families away from specialised paediatric services in regional, rural and remote locations make the use of telehealth a practical and logical choice; and 2) a collaborative relationship between the Queensland Children’s Cancer Centre and the University of Queensland’s Centre for Online Health, has been successful in securing funding over several years to support telehealth projects. The desire to improve services for children needing palliative care stemmed from previous research undertaken at the RCH, which identified that compared with families living in urban areas, families living in rural and regional areas with a child who required palliative care were disadvantaged in their ability to access appropriate care. This disadvantage was attributed to the geographical spread of families across large areas and the relatively small number of clinicians with the competence and confidence to care for a child at the end of life outside of urban areas [14]. The use of telehealth was considered by the health service to be an appropriate tool to improve access to services for families, and provide education and support for local clinicians.

**Figure 1:**
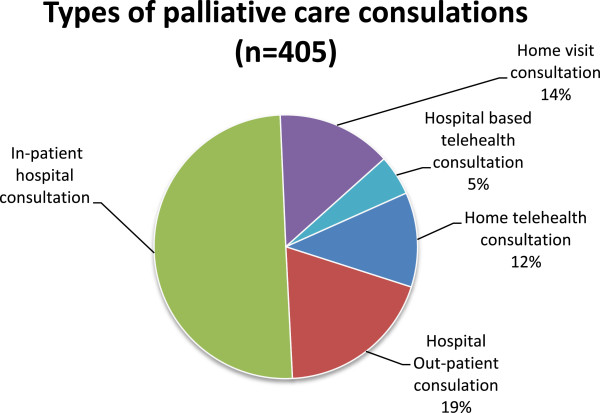
Types of consultation (n=405).

Contrary to the findings of other home telehealth studies in palliative care [11], the Paediatric Palliative Care Service (PPCS) at the RCH had expressed their willingness to embrace technology and integrate home video-consultations into routine practice. The Home Telehealth Program (HTP) was formally established in 2009 and has previously been described [15,16]. The program is used to deliver scheduled consultations with the PPCS (or other specialists) based at the RCH, directly into the family’s home, often in the presence of a visiting community nurse or general medical practitioner. The presence of primary care clinicians is an important factor in the model of care for the HTP, as the program is not intended to replace home visits, but to improve equity of access to specialist care. During home video-consultations multiple issues are discussed: symptom management; caregiver and family coping; psychological well-being; social issues including school; anticipated events; emergency plans, and practical issues such as the organisation of scripts or equipment. Video-consultations generally last for one hour and written summaries of the consultation are documented in the patient medical records with copies provided to all relevant clinicians.During a six-month time period (July- December 2009), there were 56 patients who were actively cared for by the PPCS with 39% (n = 22) residing in regional or rural locations. Over this six-month period, there were 405 consultations recorded. The HTP accounted for only 12% (n = 47) of activity, with the majority of activity related to in-patient consultations (n = 203, 50%). There were also a number of hospital to hospital based telehealth consultations; these usually occur when a patient is being transferred back to a regional hospital, or when the health team in a regional hospital request a specialist consultation. As well as consultations, during this six-month time period there were records of 1054 telephone calls and 93 email interactions with families (Figure 1).

The factors considered to be conducive to a successful program [10,17] were present, including a well-resourced program, clinical champions and receptive families. Thus, the expectation was telehealth activity would increase over time. Instead, over the years the program has been in existence, activity has decreased and is now used with only a handful of families. Therefore, the aim of this research was to determine the barriers, enablers, and perceived usefulness of the HTP from the perspective of health care clinicians, in order to establish why utilisation of the HTP was low.

## Methods

### Setting and resource requirements

The study protocol obtained ethical approval from the hospital’s ethical review committee [HREC/03/QRCH/16/AM02].

### Participant recruitment

A purposive sample of clinicians (medical, nursing and allied health) who provided palliative care for paediatric patients and who used the HTP was selected. Clinicians were approached in person or by email to discuss participation in semi-structured interviews. All clinicians were provided with a participant information sheet and written consent was obtained.

### Data collection

#### Interviews

Interviews were undertaken at a mutually agreeable time and all interviews were audio recorded. The interview structure consisted of ten base questions which were expanded upon with probing questions (see Table 1). Questions were chosen by consensus of the research team and based on a similar study in adult palliative cancer care [18]. Analysis of data commenced with data collection and all relevant issues identified in earlier interviews were incorporated into subsequent interviews, an approach used in grounded theory [19]. Audio recordings of interviews were transcribed verbatim. Transcription was verified by simultaneously listening to the audio recording of interviews and reading the transcript.

**Table 1:** Semi structured interview guide

**Interview questions**	**Checklist and further questions**
1. What do you consider to be the most important interventions to support a child receiving palliative care and their family at home?	Starting point- establish rapport, identify areas for further probing questions:
	Relevant situations
	Previous involvement with the HTP
2. Do you think there is a role for home video-consultation in the care of these families?	Can you explain why?
3. Have you seen home video-consultation used in the care of these families?	Are you able to give an example?
4. Do you think there are any benefits of using home to support families?	Can you describe benefits you have seen?
5. Do you think there are limitations using home video-consultation in the care of these families?	Can you describe the limitations you have come across?
6. Do you think communicating via home video-consultation changes your relationships with families?	Can you explain why/why not?
7. What do you think the barriers are to home video-consultation in the care of families?	Are you able to give an example?
8. How satisfied are you with using home video-consultation for palliative care?	Can you explain why?
9. What factors influence your decisions regarding your form of communication with families at home?	Identify relevant factors applicable to the HTP and probe further
10. What would you change to improve services for families at home caring for a child receiving palliative care?	Identify relevant situations for further probing. Offer opportunity to discuss any other relevant issues. Close.

### Data analysis

The first two interviews were initially indexed and preliminary analysis undertaken by hand. Subsequently, the computer software program N-Vivo 9 was used to apply thematic indexing consistently to the transcriptions of all interviews. Indexing data involved using a consistent system according to common principles and measures [20]. Each code initially formed a potential category, but as indexing progressed, like categories were grouped together to form themes. Themes and categories were also developed within the context of the research question, in order to identify the barriers, enablers and perceived usefulness of the HTP. As the analysis progressed, the process of moving back and forth between the indexed data and the original transcripts was used to ensure the themes were representative of the intended meaning of participants [21]. Each theme was examined across all participants, the range of perceptions was noted, and illustrative quotes were extracted. As the core concepts emerged from the data, a theory was generated to describe how concepts related to each other and could inform the established research aims.

## Results

### Participants

Ten clinicians were approached to participate in the interviews, all of whom provided consent and completed a semi-structured interview. Table 2 presents the professional discipline of the clinicians. Interviews lasted between 26 and 38 minutes (mean 32 minutes). All participants were experienced clinicians who had worked on the Paediatric Palliative Care Service or in the Paediatric Oncology ward for over five years (tertiary, metropolitan referral centre), and who had experience with using the HTP.

**Table 2:** Professional background of participants

**Discipline**	**N**
Medical	4
Nursing	5
Allied Health	1
*Total*	*10*

### Findings

A summary of the barriers, enablers and perceived usefulness of the HTP are presented in Table 3. Four themes emerged from the analysis: i) managing relationships; ii) expectation of clinicians; iii) co-ordination, and iv) the telehealth compromise. Following discussion of these themes, the theory which evolved to explain the telehealth compromise is discussed. A conceptual model of the core concepts of control and trust is presented.

**Table 3:** Clinician perspectives of the barriers, enablers and perceived usefulness of the home telehealth program

**Barriers**	**Enablers**	**Perceived usefulness**
**Technology factors**		
Limited or inconvenient access to equipment	Families who have access to required technology. Suggestion of having equipment in more convenient locations for clinicians, e.g. from their PC or mobile devices	Having easily accessible equipment reduces the ‘hassle’ of participating in a video-consultation
Burden of setting up families with equipment, usernames and passwords at a stressful time	Families who are familiar with video communication and have access to the required technology	Simple to set up if family familiar with technology and a consultation can occur rapidly without difficulty
Comparative ease of telephone use	Clear benefit of using video, e.g. to observe a wound, or breathing pattern	Provides visual information not available in a telephone call
Discomfort with using technology	Previous experience or a willingness to participate in video-consultations	
Privacy concerns- unable to control home environment, concerns with using the Internet	Having sound proof studios where video-consultations can be undertaken without interruption within the hospital	Ability to include multiple members of the health care team means information can be shared during one conversation
**Individual factors**		
Personal preference for face-to-face interaction, video-consultations not a suitable substitute	Receptive families who request ongoing home video-consultations. Supportive local clinicians who are willing to participate	Presence of community-based clinicians enables ensures human presence available at family end
Cultural, linguistic, socio-economic diversities may make communicating via Internet-video difficult	Immigrant families often more familiar with using Internet-video to communicate with family oversees and may be more receptive to receiving health services via home video-consultation	Ability to include multiple family members in a consultation, e.g. Indigenous Australians often leave important decision making to the tribal elder not the parents or caregiver of the child
**Service factors**		
Establishing routines	Having a coordinator to schedule video-consultations and manage administrative issues	Efficient process of communicating with multiple stakeholders
Strengthened community support: reduced need for video-consultations with PPCS	Partnering with COH ensures clinicians can remain focussed on clinical care not managing telehealth	Facilitates provision of peer-peer support and education
Lack of time; focus on hospital inpatients	Suggestion that having routine clinics for home video-consultation may be easier to manage than ad hoc	Ability to provide a consultation across vast distances which would otherwise require many hours of travel time
Staff shortages		

### Managing relationships

Families establish strong relationships with their specialist teams based at the tertiary hospital [22] and despite community teams providing primary care, participants reported that families continue to value the input of the specialist team. The interviews highlighted that specialist clinicians assume the responsibility for maintaining these relationships and ensuring that families perceive they are cared for.

“Contact with the family (is needed) in whatever shape or form…and the contact needs to be fairly consistent so the families don’t feel abandoned…that makes a huge difference in their trust.. that you will be there for the whole journey, that you are looking out for them.” (Nurse)

Providing families with choice in how they engage with the team and what services they accept, was seen as a positive process that enabled families to ‘steer’ their relationship with the team. The concept of providing choice and flexible care that is responsive to a family’s needs is a theme evident in the literature [23,24]. It was discussed there was a need for an individual approach when planning any intervention or interaction; control of the relationship between clinicians and families was guided by needs of the family but it was the actions of the clinicians that determined how needs were met. The HPT was reported to offer an alternative mechanism to maintain relationships with families in regional or rural locations that was “in between” a phone call and a face-to-face consultation.

“You can still do all those conversations by the phone, but [if] we can’t physically go out there and visit then, you know, you can eyeball the child and if the parents are talking about changing colour or going jaundice, then you can see that.” (Medical Doctor)

The participants discussed how the HTP was able to facilitate a greater appreciation of the individual needs of families and enhance therapeutic relationships when challenged by distance compared to communication by telephone. It is not known whether this enhanced relationship is also felt by families, or whether this is a perception of clinicians only, but this appreciation of the ‘identity’ of the family supports the concept of family centred care:

“I think it gives us as a team greater insight into…[the family] it’s like a home visit…whenever I do a home visit it reminds me of the place of that child in their community and the family. Whereas when it’s a phone conversation, for whatever reason, I don’t have that same experience.” (Social Worker)

“You know you can see a bit of the background and they might flick the camera around and you get a greater sense of their home and it’s like you’re being invited in more closely.” (Nurse)

The HTP was thus generally considered a means of enhancing communication and maintaining relationships, and particularly for case conferences where multiple individuals were present, the HTP was seen as a time efficient process for ensuring all relevant parties could be updated and included in discussions. Conversely however, clinicians were also cognisant of the potential emotional distress that may occur in a consultation, for example when discussing deterioration or end of life care. For these sensitive topics, clinicians held some hesitancy regarding the appropriateness of telehealth.

There was some discussion of the appropriate duration for a home video-consultation and the need for the right balance of social interaction and clinical discussion to occur in a home setting, compared to the predominantly clinical discussion which occurs in a facility:

“We’re being invited into their own personal space, and so we’re always cognisant that we actually are in their home. And that’s quite a privilege I think, so it does actually bring you closer; partly because you’ve been invited you to come into their environment.” (Social Worker)

One participant discussed how a planned telehealth consultation was closer to a face-to-face consultation in terms of the comprehensive nature of discussions held via telehealth compared to telephone. The extra preparation and desire to undertake a thorough consultation was evident:

“When you actually schedule in a teleconference, as opposed to just talking to the family on the hop, you can be more thorough and put more thought into what you are suggesting, you know maybe do a lot of surveillance or anticipatory screening for symptoms, whereas maybe if you are just talking to the family on the phone, you are just dealing with the issue at hand.” (Medical Doctor)

The issue of privacy was described as a barrier to the HTP in various contexts. Others being able to hear the conversation in both the hospital and the home setting challenged the importance of being able to conduct sensitive conversations privately. The home environment is difficult to control; the location of equipment and presence of other family members not normally included in consultations was reported to effect privacy. It was also discussed that on occasions families appeared overwhelmed if there were too many clinicians involved in a video-consultation; consideration needs to be given as to who should be included in consultations. Additionally, the views of the older child were also considered by participants. As children move towards adolescence, their autonomy and independence is threatened by their illness, they may resent any intrusion into their personal space as indicated by this participant

“You know some are of the age where they don’t allow even mum or dad into their rooms so the idea of someone spying on you though the computer maybe not helpful when you are not feeling your best.” (Nurse)

Despite these issues the HTP was generally seen as a valuable tool for managing relationships. However, the poor utilisation of the HTP indicates other factors override these benefits and impede use.

### Expectations of clinicians

There was unanimous support for the program, with clinicians stating the program was valuable, should routinely be used for patients, and that families should be encouraged to use telehealth more. The contradiction between the expectations of the HTP to be a useful tool and the observed poor utilisation was therefore highlighted. This may be explained by the difference between expectations and the experience of actually providing care to families through this medium.

While the ability of the HTP to maintain continuity of care was raised by a number of interviewees, there was also the suggestion that the HTP offered ‘too much’. The ability to provide a consultation right into the family home created the potential of over-servicing and overloading families with information. The notion of trying to address every issue all at once, and engaging other clinicians in a consultation when they may not be required was evident in several interviews. It appeared the balance required between what clinicians believed should be provided as a service and what families actually required was not well defined. While clinicians hinted this was an issue, this point was not elaborated further during the interviews.

Moreover, the expectations from some clinicians that the HTP should be used as a default service, used with all families in regional and rural locations, contradicted the premise of providing individualised care and ignored the issue of the suitability of the HTP for individual families. It appeared clinicians believed they *should* be using the HTP; it was something they had wanted after all, but there was a reluctance or inability to articulate why the HTP was not used more often. Despite pilot work in this area since 2004, several participants stated it was something they just needed to get used too; it was still ‘new’ and not yet integrated into clinical practice.

As well as supporting families, it was evident in the interviews that specialist clinicians held the expectation that they should also support the primary care clinicians who provided the day-to-day care for patients and families in their local communities, and that telehealth was a logical option for doing so. The practice of scheduling video-consultations when the community nurse or general practitioner was home visiting was reported to be a time efficient method of transferring information and providing peer-to-peer support and informal education to health care providers who may not be experienced in caring for a child and their family towards the end of life.

“They really find that connection helpful. And they get to hear what you have to say and how you speak to the family and you can guide them by remote control.” (Nurse)

### Coordination

The co-ordination aspects of the HTP were acknowledged by several participants. Having all aspects of administration and coordination managed by an external source was reported to help clinicians concentrate on providing clinical care. However, there was concern that the HTP was not offering an equitable service; while the purpose of the HTP is to improve access to specialists, this requires families have the necessary technology. Over the last decade, the numbers of families who have access to computers and the Internet has increased dramatically. However, families without the resources required remain, and these families need special consideration and equipment loaned, which potentially creates additional burdens at a stressful time.

Additionally, despite removing the administrative and technical issues from the clinician’s responsibility, the HTP required clinicians to consider and modify the way they practice and manage patient care.

“Sometimes if I am worried about a patient I will simply pick up the phone. I can’t do that on telehealth at this point in time. There is no reason that couldn’t happen, apart from the logistical set-up. But probably there is the matter of how you think about it, the matter of mind. I think that, the trends that we have socially, that will drive all of that.” (Medical Doctor)

“We see a lot of families who don’t have furniture let alone a computer, and so running around worrying about computers and Internet is an added drama for them when they are worried about the sick baby or the sick child.” (Nurse)

“It doesn’t take long from this end but of course also the family need to turn their computer on. And sometimes the family may not have theirs set up where it’s in a convenient location. If they’ve got a laptop its fine but if they’ve got a PC, it may be in an office down the end of the house and the sick child is at the other end.” (Medical Doctor)

Other co-ordination issues discussed by the participants centred on the PPCS itself and how services are delivered. One of the activities of the PPCS, since its establishment in 2009, has been to co-ordinate home care through referral to domiciliary services. Strengthening this network of service providers has resulted in less reliance on the PPCS to deliver some aspects of care. As the community capacity to manage the day-to-day responsibility of patient care has increased, the actual need for PPCS involvement and thus a home video-consultation is reduced. While a ‘barrier’ to the HTP, the benefits to the patients, families and wider community are a desirable outcome.

“Look I think, maybe one of the reasons [HTP is not used], is because the communication between the local health providers and the families is actually so well done, that they may not necessarily find they have the need for it.”(Nurse)

“If a patient’s going home we ensure that there is a domiciliary agency linked in, that there’s a regional paediatrician or therapists that are present so that we’ve encouraged them to look after the family. So it may be that we’re doing too good a job. And I would hope that that’s the case.” (Medical Doctor)

Other service driven issues included time shortages and pressures on staff to attend to patients who are admitted as inpatients to the hospital, limiting time available to consider families in the community.

### The telehealth compromise

The use of telehealth can require a compromise. While the interviewees confirmed telehealth is regarded as an efficient, cost effective, feasible way of providing care, that improved equity of access to care, it was also acknowledged it is inferior to in-person interactions and not the preferred mode of communicating with patients. Traditional models of care rely upon human presence to deliver palliative care and it was not clear from the interviews if the clinicians felt they could deliver the same service with a virtual presence. While the HTP offered the desired possibility of providing access to specialist care regardless of location, participants indicated they were not always confident they could deliver the required care using telehealth. Technology sometimes fails; interrupted consultations are undesirable outcome for both clinicians and families.

“It’s not one hundred per cent reliable. And, you know I’ve had a number of experiences where we’ve been liaising with families by webcam and by the time we get to the psychosocial, for whatever reason, the technologies decided to fail. And that’s really frustrating. I think for everyone.” (Social Worker)

Additionally in times of high stress and the busyness of a clinical workload, the HTP was viewed negatively as ‘one more thing that needed to be done’ rather than an efficient method of providing clinical care. These factors may explain the poor uptake of the service.

“I know it’s not hard…and I have to get past that too…picking up the phone seems so much easier, and I can do that on the run between other jobs.” (Nurse)

As well as the factors related to technology, participants reported individual preferences for communication. Two participants cited examples of caregivers being ‘camera shy’ and not wanting to appear on camera; the difficult and sometimes distressing conversations that occur during interactions with caregivers are not necessarily made easier by having visual communication.

“There was one time when a mum got teary and was crying ‘I am sorry, I am sorry’ and had to physically walk away from the camera because she felt that, was you know, ‘you can see that I am crying’. And that was a hard thing to manage at that time, because you’re not there in person and you can’t actually comfort the person. And I think if that was over the phone I don’t think it would have been as confronting for that mum and I think the conversation would have rolled on a bit better.” (Nurse)

“I think barriers from the family perspective may well be the intrusion into their environment and some of my families previously have actually not wanted to have the video in their home. They much prefer to have telephone conversations so they can actually be outside of the house [out of earshot from the patient] even to have a simple conversation.” (Nurse)

Experiences, like the examples described, are likely to influence both the clinician’s and the caregiver’s perception of the usefulness of a video-consultation and highlight the need for there to be a clinical indication for video communication. If human presence is not possible, it may be that virtual presence is not an acceptable alternative.

Thus exists the compromise; a tension between conventional methods of providing care, which relies on physical presence, and the possibility of increased access to care which involves a virtual presence with the associated limitations. The theory which developed to explain this compromise further is discussed below.

### Theory development

The core concepts that emerged from the data were control and trust. When combined with barriers or enablers, these two concepts encapsulate the phenomena of the HTP from the perspectives of the clinicians who use the program. The need to depend on technology to facilitate an interaction may require control of the interaction to be somewhat relinquished because of the following factors:

•Limited visual field: not being able to see the whole environment the family is situated in, and the limited visual field of a screen, may hinder communication in some instances. The two-dimensional visual images made possible with a video-consultation may impede a clinician’s ability to interpret family needs and provide appropriate care. Interpreting the images seen during a home video-consultation may therefore be a skill that needs to be developed by both the clinician and family at home.

•Flow of communication: compared with telephone or in-person communication, communicating via video can be less spontaneous and free flowing, impeded by unnatural pauses and delay. This can result in a consultation feeling awkward and a clinician may feel they haven’t quite been able to direct the consultation the same way they would have in a face-to-face consultation.

•Lack of presence: during a video-consultation, the ability to use touch as a form of communication is eliminated. The patient and family is no longer *here*…but *there*; the perception of being present is altered. The loss of human presence and ability to use touch as a form of communication when using telehealth may leave clinicians without a form of communication they are familiar with in their routine practice. This may affect their perception of their ability to control the consultation.

•Ability to convey empathy and compassion: these values are inherent in the delivery of palliative care. Little is understood about how to convey these values in the virtual environment and how these aims may be achieved. While a significant amount of work has been undertaken with telepsychiatry [25-27], the knowledge gained in this specialty does not readily transfer to palliative care, or to home care. Video-consultation may actually be viewed as an intrusion into a family’s home at a time when they feel most vulnerable.

•Alteration of social categories: In a traditional consultation, the clinician is ‘in charge’. In a home consultation, this is reversed, the family’s environment is respected and clinician is invited into that space. In a video-consultation, it may not be clear who is ‘in charge’; this may not be desirable for either the clinician or the caregiver. The change in power dynamic between the clinician and caregiver can leave both parties uncomfortable with feeling the consultation wasn’t as ‘good’ as it could be.

**Figure 2:**
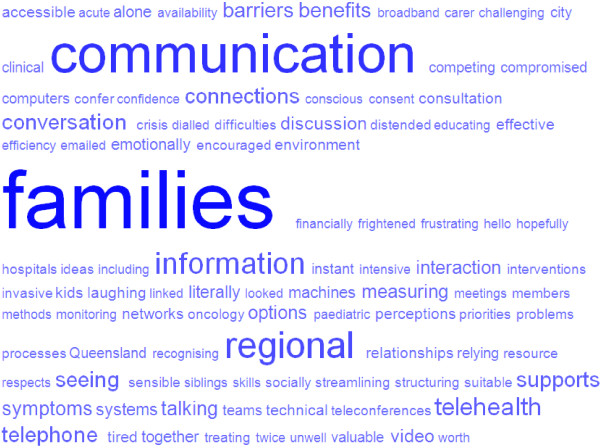
Tag Cloud of word frequency from interviews.

Relying on the HTP for communication requires surrendering the elements of control clinicians (and families) have in face-to-face interactions and to trust that they will be able to communicate effectively in the virtual space of a video-consultation.A visual ‘tag cloud’ representation of the frequency with which words were used in the interviews is presented in Figure 2 (See tag cloud). Words with higher frequency count are in larger text. This visual representation can be used to quickly identify the key words and prominent themes within interview data. The words ‘families’ and ‘communication’ are the largest words in the tag cloud, representing the most common words found within the transcripts. It is clear the central foci for paediatric palliative care are the families and the desire for clinicians to keep the channels of communication open and effective. While telehealth presents an option for undertaking this, clinicians may be uncomfortable with trusting technology and their ability to control and manage conversations in the virtual environment of a telehealth consultation.

## Discussion

Good communication between caregivers and clinicians is recognised as a core value of palliative care [24,28,29]. The use of video-consultation to support families who live outside of urban areas, where access to specialist care is limited, presents an option for facilitating real time communication between palliative care services and families who are not able to have a face-to-face consultation. In this study a home telehealth service, which was established in 2009, was investigated. Clinician perceptions of the barriers, enablers and usefulness of a home telehealth service for paediatric palliative care were identifed. The barriers to the HTP described by participants in this study were consistent with barriers reported in other studies of telehealth applications [5,11], however, there were also some new findings not previously reported. While there was acknowledgment that some clinicians found the technology daunting or cited some other barrier for low utilisation, all clinicians believed these were barriers that would be overcome with time, once the program was fully integrated with the service. This has not been observed; utilisation has continued to decline over time rather than grow. Given the complexities of care and the multiple other interventions required, it may be that utilisation of the program is appropriate for only a small number of patients. Potentially, the original expectations for use may have been too high. As the PPCS has grown and developed as a service, it has established a network of primary care clinicians in the community, and through education, has increased their capacity to provide direct care. Thus it may be there is a reduced requirement for the PPCS to be directly involved with families and therefore less need for home telehealth in this specific population.

Across the world, interest and resources continue to be directed towards home telehealth programs in palliative care. Nagel and colleagues [30] discussed the contradictory aspects of technology which can bridge service delivery gaps, but at the same time create a physical distance and a perception of losing human presence. There is a requirement for further understanding and acknowledgment of the compromise that exists when palliative care consultations are delivered via telehealth; clinicians and families need to agree that the compromise is acceptable, and that the benefits outweigh the loss in human presence. Some clinicians may feel they are less able to control situations when they can see but not touch or offer human presence, and clinicians require confidence to communicate in the virtual environment to manage difficult conversations.

However, there may be many situations where the compromise is acceptable and for those cases the use of telehealth should be supported, encouraged and facilitated. Indeed to not embrace the ability to provide a home video-consultation in today’s advanced technological era could be viewed negatively. There is a growing expectation from governments, consumers and society that technology should be used to improve access to care, and there are increasing numbers of families who are comfortable with technology and willing to engage with health care providers using this medium. The ability of the home telehealth to address the disparities of access to specialised care for those living in rural and remote locations should not be underestimated.

### Limitations

There are several limitations with this study. A relatively small sample size was used, and the interviewer (NB) had previously worked with and knew the interviewees. This may have biased the process of data collection. However, this relationship also helped to build rapport and trust during the interview process. Other types of evidence are required to fully understand this problem, and it would be helpful to understand this issue from the perspectives of the families also; those who used the HTP and those who did not.

## Conclusion

Video-consultation into homes has the potential to provide a solution to inequity in access to care, facilitate peer support and maintain continuity of care with families. The interviews highlighted important areas to consider when determining interventions and methods of communication with families when a child is receiving home based palliative care. The HTP is viewed positively by the clinicians who care for children receiving palliative care and as a useful method to provide ongoing support and clinical care. Use of the HTP however was reported to be limited by several factors including technology, time and personal preference (of either the clinician or family) for face to face or telephone only support. Additionally clinicians need to trust their ability to control a consultation in the virtual space which they may be unfamiliar, or uncomfortable with. Further education and research is required to better understand how to communicate in this virtual space, including how to effectively convey empathy and compassion.

## Competing interest

The authors declare that they have no competing interest.

## Authors’ contribution

NB collected, analysed and interpreted data, and drafted the manuscript. JY analysed and interpreted data and revised the manuscript. AH conceived of the study design, participated in its design and coordination. NA participated in the coordination of the study and revised the manuscript. AS interpreted the data and revised the manuscript. All authors read and approved the final manuscript.

## Pre-publication history

The pre-publication history for this paper can be accessed here:

http://www.biomedcentral.com/1472-684X/13/29/prepub
